# Transdifferentiation of Myoblasts Into Adipocytes by All-*Trans*-Retinoic Acid in Avian

**DOI:** 10.3389/fcell.2022.856881

**Published:** 2022-04-06

**Authors:** Dong-Hwan Kim, Joonbum Lee, Yeunsu Suh, Jae-Kyun Ko, Kichoon Lee

**Affiliations:** ^1^ Department of Animal Sciences, The Ohio State University, Columbus, OH, United States; ^2^ The Ohio State University Interdisciplinary Human Nutrition Program, The Ohio State University, Columbus, OH, United States; ^3^ Department of Surgery, Davis Heart and Lung Research Institute, The Ohio State University, Columbus, OH, United States

**Keywords:** transdifferentiation, myoblast, adipocyte, all-trans retinoic acid, Ppar gamma, avian

## Abstract

Increased adipogenesis in muscle tissues is related to metabolic syndromes and muscle weakness in humans and improvement of meat quality in animal production. With growing evidence for pro-adipogenic functions of all-*trans*-retinoic acid (atRA), the current study investigated whether atRA can transdifferentiate myoblasts into adipocytes using a quail myogenic cell line (QM7) and avian primary myoblasts. atRA increased cytoplasmic lipid droplet accumulation and mRNA expression for adipogenic genes in these cells. An acute induction of Pparγ expression by atRA under cycloheximide treatment indicated a direct regulation of Pparγ by atRA. In addition, the induction of Pparγ expression was mediated by retinoic acid receptors . At high levels of Pparγ by atRA, BADGE, an antagonist of Pparγ, inhibited, and rosiglitazone, an agonist of Pparγ, further enhanced atRA-induced transdifferentiation. However, at very low levels of Pparγ in the absence of atRA treatment, rosiglitazone could not induce transdifferentiation of avian myoblasts. These data suggest that the induction of Pparγ expression by atRA is an essential molecular event in myoblasts for atRA-induced transdifferentiation into adipocytes. Based on our findings, atRA can be a new transdifferentiation factor of myoblasts to adipocytes, providing a potential nutrient to enhance marbling in poultry.

## 1 Introduction

Intramuscular adipose tissues (IMAT; also termed as marbling fat) are found within muscles. In meat animals, the amount of IMAT is an important criterion to decide the meat quality including flavor, tenderness, and juiciness (Albrecht et al., 2006). Similar to ruminants, including cattle and sheep, amounts of IMAT in poultry breast are generally related to juiciness and tenderness suggesting an importance of IMAT in poultry meat quality (Lonergan et al., 2003). Therefore, identification of new factors increasing IMAT and understanding of their functions lead to developing potential strategies for enhancing marbling fat and improving meat quality in poultry.

Adipogenesis is a process of converting mesodermal precursor cells into adipocytes (Farmer 2006), being accompanied by the initiation of expression of adipogenic transcription factors including peroxisome proliferator-activated receptor γ (Pparγ). These precursor cells have abilities to be differentiated into myocytes with the expression of myogenic transcriptional factors (Rosen and MacDougald 2006; Rodeheffer et al., 2008). Especially, Pparγ has been served as a well-known indicator for adipogenic differentiation in mammals (Tamori et al., 2002; Liang et al., 2016) and avian species (Kim et al., 2020a, Kim et al., 2020b, Kim et al., 2021a, Kim et al., 2021b; Lee et al., 2021). In addition, over-expression or activation of Pparγ can convert myogenic cells to adipocytes (Hu et al., 1995; Teboul et al., 1995). Supplementation of vitamin D in adipogenic media induced transdifferentiation of the C2C12 myogenic cell line to adipocytes, which is accompanied with the expression of Pparγ (Ryan et al., 2013). Pparγ has served as an important differentiation marker and a potential inducer for transdifferentiation of myocytes to adipocytes in cultures of rodent myogenic cell lines.

All-*trans*-retinoic acid (atRA) which is as a metabolite of vitamin A (retinol) functions as a regulatory molecule in cell differentiation and development including adipogenesis both *in vitro* and *in vivo*. Previous studies showed that adipogenic differentiation can be induced by the supplementation of atRA. Ob1771 cells were differentiated into adipogenic cells by the supplementation of 10 nM of atRA (Safonova et al., 1994). Adipogenic differentiation of 3T3-L1 cells was negatively or positively regulated by supplementation of high- or low-doses of atRA, respectively (Kim et al., 2019). In various avian cell types, chicken embryonic fibroblasts which are isolated at embryonic day (E) 5, stromal vascular cells isolated from fat tissues of chicken embryos, and a DF-1 chicken embryonic fibroblast cell line can be induced to adipogenic cells in the culture condition supplementing atRA (Kim D.-H. et al., 2020; Lee et al., 2021). Furthermore, direct *in ovo* injection of atRA increased adipose weight and size during embryonic development (Kim et al., 2021a). Although atRA has positive regulatory effects on adipogenic differentiation of various avian cell types, it has not been specifically investigated whether atRA can induce transdifferentiation of myoblasts into adipocytes in poultry. In this study, a quail myogenic cell line (QM7) and primary myoblasts from chicken and quail embryos were used to investigate the effects of atRA on transdifferentiation from myogenic to adipogenic cells and the involvement of Pparγ in this process.

## 2 Materials and Methods

### 2.1 Induction of Adipogenic or Myogenic Differentiation

The quail muscle clone 7 (QM7, ATCC, #CRL-1962, Rockville, MD, United States) cell line was cultured in medium 199 supplemented with 10% fetal bovine (#F4135, Sigma-Aldrich, St. Louis, MO, United States), 10% tryptose phosphate broth (#T8159, Sigma-Aldrich), and 1% antibiotic-antimycotic solution (#15240062, Gibco, Grand Island, NY, United States). Primary myoblasts were isolated from pectoralis muscles of each quail embryo at E12, and each chicken embryo at E13, totaling eight embryos per species in four independent experimental trials, as followed from our previous study (Hassan et al., 2014). Muscle tissues were digested in DMEM containing 1.6 mg/ml collagenase II (#17101015, Sigma-Aldrich) at 37°C with a shaking incubator for 1 h. Then, the tissues were filtered through a 70 mm cell strainer (#352350, BD Falcon, Ahn Arbor, MI, United States) and seeded on a collagen (#A1048301, Thermo Fisher Scientific, Waltham, MA, United States) coated 12-well plate after washing with PBS. The primary myoblasts were cultured in Dulbecco’s modified Eagle’s medium (DMEM, #11965, Gibco) supplemented with 10% fetal bovine serum (FBS, #F4135, Sigma-Aldrich), and 1% antibiotic–antimycotic solution (#15240062, Gibco). To test the potential effect of atRA on adipogenic differentiation of myoblasts, different concentrations of atRA (0 μM, 100 μM, 150 μM, or 200 μM, #R2625, Sigma-Aldrich) were supplemented to the basic adipogenic media, DMEM containing only 10% chicken serum (CS, #C5405, Sigma-Aldrich) (Kim et al., 2021b), for 48 h. Due to similar viability of the cells between the two groups, 0 and 100 μM of atRA (Supplementary Figure S1), 100 μM of atRA was mainly used in this study. Myogenic differentiation was induced by 1% horse serum (HS, #16050, Gibco) for 48 h.

### 2.2 Lipid Droplet Formation Assay

To examine lipid accumulation, after inducing adipogenic differentiation of the cells for 48 h, they were stained by Oil-Red-O (ORO) to visualize and quantify the formatted lipid droplets as followed from our previous study (Kim D. H. et al., 2020). For quantifying lipid accumulation, ORO was extracted with 100% isopropanol and absorbance values were measured at 490 nm by a spectrophotometer (SpectraMax Plus384, Molecular Devices, Sunnyvale, CA, United States). Undifferentiated cells were used as a negative control. Stained cells were visualized using a microscope (EVOS cell imaging system, Thermo Fisher Scientific).

### 2.3 RNA Isolation and Reverse-Transcription Quantitative PCR

Total RNA was isolated using TRIzol (#15596026, Invitrogen, Waltham, MA, United States) and cDNA was synthesized by Moloney murine leukemia virus reverse transcriptase (#28020513, Invitrogen) by the methods described in our previous study (Kim D. H. et al., 2020). qPCR was conducted by using AmpliTaq Gold polymerase (#N8080241, Applied Biosystems, Foster City, CA, United States) and SYBR green as detection dyes on an ABI 7300 Real-Time PCR instrument (Applied Biosystems). To quantify gene expression levels involved in adipogenesis and myogenesis, over three independent experiments were performed and each experiment was duplicated. qPCR was performed in duplicate with specific primer sets (Supplementary Table S1). The expression levels were normalized to those of endogenous glyceraldehyde-3-phosphate dehydrogenase (Gapdh) and the data were analyzed using the ddCt method (Livak and Schmittgen 2001).

### 2.4 Chemical Treatments

All chemicals, atRA, AGN194310 (AGN, #SML2665, Sigma-Aldrich), cycloheximide (CHX, #C7698, Sigma-Aldrich), bisphenol A diglycidyl ether (BADGE, #D3415, Sigma-Aldrich), and rosiglitazone (Rosi, #R2408, Sigma-Aldrich), were dissolved in dimethyl sulfoxide (DMSO). In order to examine the acute changes of expression levels of Pparγ after supplementation of atRA in myogenic cells, chicken or quail primary myoblasts, and QM7 cells, the cells were incubated with different concentrations of atRA (0 μM, 150 μM, or 300 μM) for 0, 3, or 5 h and then, qPCR was performed to analyze the expression levels of Pparγ.

To further investigate whether a new protein translation is required for the induction of Pparγ expression by atRA, 10 mg/ml of CHX, an inhibitor of protein synthesis, was pre-incubated for 1 h and then, after washing with PBS, further incubated with a medium supplemented with different concentrations of atRA (0 μM, 150 μM, or 300 μM) for 5 h. After 5 h, the expression levels of Pparγ were analyzed from each of the cells supplemented with atRA. In addition, to examine if the induction of Pparγ by atRA can be mediated by retinoic acid receptors (RAR), different concentrations (0 μM, 10 μM, or 100 μM) of AGN as an RAR antagonist were supplemented with or without 150 μM of atRA for 5 h in QM7 cells.

BADGE or Rosi, an antagonist or agonist of Pparγ, respectively, was used to investigate the effects of Pparγ activities on atRA-induced transdifferentiation. One or 2 mM of BADGE was co-incubated with 100 μM of atRA, and Rosi (10 nM) was co-incubated with 50 μM of atRA during the induction of adipogenic differentiation for 2 days. At D2 of differentiation, the cells were stained with ORO.

### 2.5 Statistical Analysis

All data in this study were replicated at least three times (*n* ≥ 3) and were expressed as means ± SEM. All statistical analyses were performed by the GraphPad Prism software (ver. 6.02) and detailed numbers of samples were described in each of the figure legends. For *t*-test and one-way ANOVA followed by Tukey’s multiple comparison test, *p*-value, *p* < 0.05, was considered as a statistically significant difference.

## 3 Results

### 3.1 Effect of atRA on Lipid Accumulation in QM7 Cells

**FIGURE 1:**
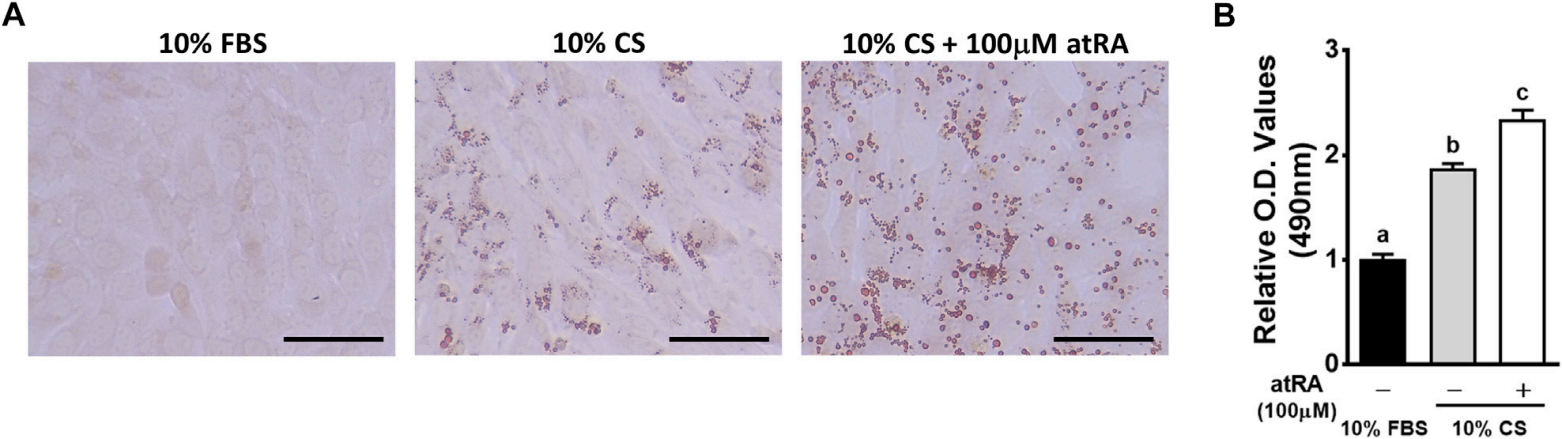
Effects of various media on the lipid accumulation in QM7 cells. Oil-Red-O (ORO) staining **(A)** and O.D. values **(B)**. QM7 cells were incubated with different media, 10% fetal bovine serum (FBS), 10% chicken serum (CS), or a combination of 10% CS and 100 μM all-*trans*-retinoic acid (atRA) for 48 h. Lipid droplets in the cells were visualized under a microscope after ORO staining and quantified by a relative amount of ORO per cell which is normalized by a group of 10% FBS. Scale bar: 100 μm. All data were shown as mean ± SEM (*n* = 8 from 4 independent experiments). One-way ANOVA followed by Tukey’s multiple comparison test was used for statistical analysis by the GraphPad PRISM program and statements of significance noted by **(A-C)** are based on testing at *p* < 0.05.

In general, insulin, isobutylmethylxanthine (IBMX), and dexamethasone have been widely used for adipogenic differentiation in a mammal system including 3T3-L1 cells. However, we developed a minimal adipogenic media containing chicken serum alone that can differentiate avian embryonic fibroblasts into adipocytes (Kim et al., 2021b). This minimal adipogenic media for avian species can be used to directly test hormonal and nutritional factors without complex interactions of several factors included in so-called adipogenic cocktails. Using the basal media containing 10% CS, adipogenic potential of atRA has been demonstrated in primary embryonic fibroblasts and a fibroblast cell line in avian systems (Kim D.-H. et al., 2020; Lee et al., 2021). In the current study, the capability of atRA inducing transdifferentiation of myoblasts into adipocytes was also tested in the basal avian adipogenic differentiation media containing CS alone. Therefore, QM7 cells were incubated in 10% CS with/without 100 μM of atRA for 48 h to induce adipogenic differentiation. Morphological and quantitative assessments of lipid droplet formation and lipid accumulation, respectively, were performed by analyses of ORO staining. The degrees of lipid droplet formation were higher in the order of 10% CS with 100 μM atRA, 10% CS with 0 μM atRA, and 10% FBS with 0 μM atRA (Figures 1A,B). Also, the quantification of ORO by spectrometrical analysis revealed a significantly increased lipid accumulation by supplementation of atRA in 10% CS (Figure 1B). These results suggest that atRA induces lipid accumulation in quail myogenic cell lines.

### 3.2 Regulation of Adipogenic/Myogenic Fators by atRA

**FIGURE 2:**
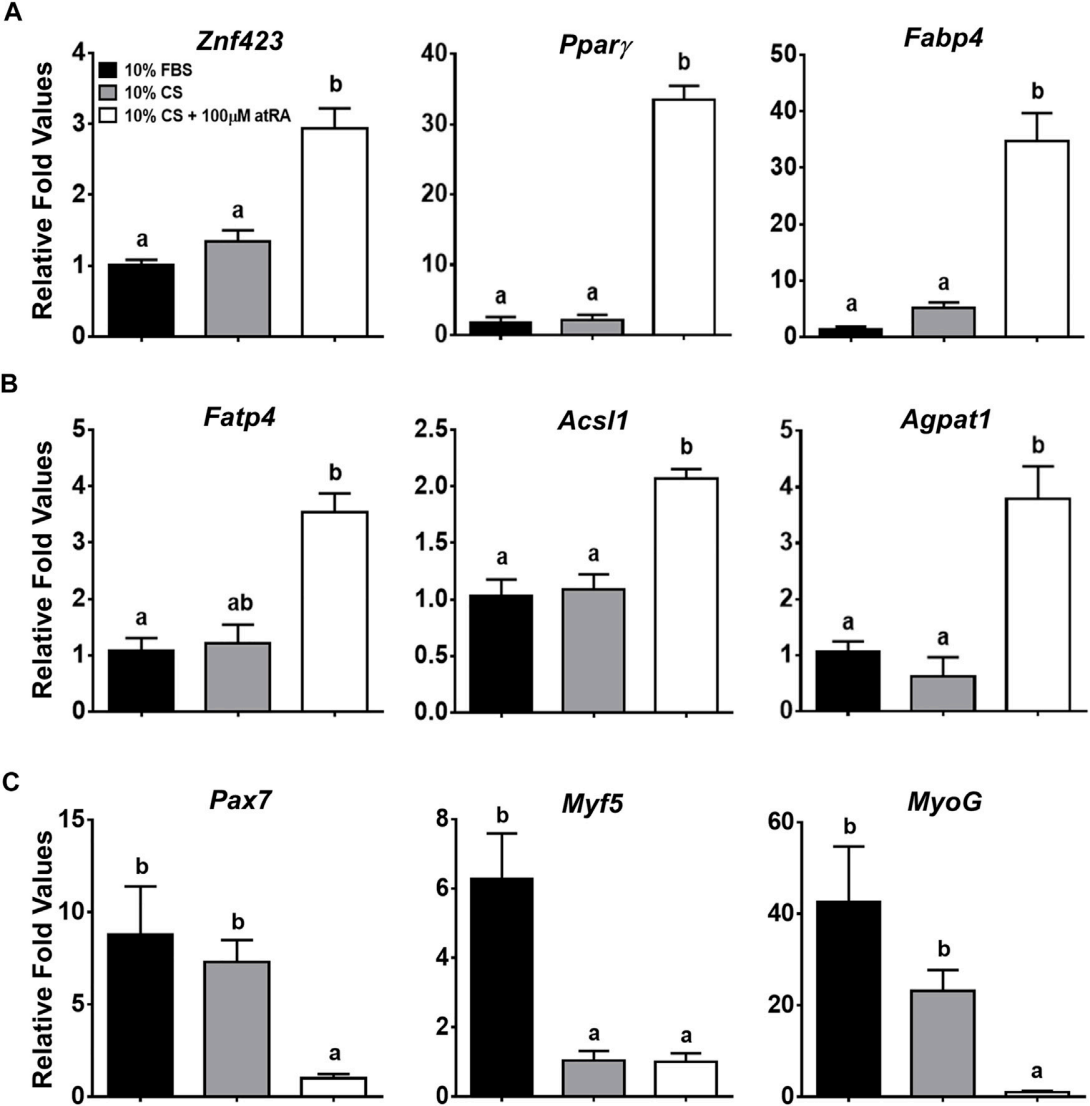
Relative gene expression levels in QM7 cells by quantitative real-time PCR (qPCR). Expression levels of genes involved in adipogenesis **(A)**, fatty acid uptake and triglyceride (TAG) synthesis **(B)**, and myogenesis **(C)** were analyzed at 48 h after incubating with three different media, 10% FBS, 10% CS, or 10% CS with 100 μM atRA. Gapdh was used as a housekeeping gene and all expression levels of each gene were normalized by the levels of a group of 10% FBS. All data were shown as mean ± SEM (*n* = 4 from 4 independent experiments). One-way ANOVA followed by Tukey’s multiple comparison test was used for statistical analysis by the GraphPad PRISM program and statements of significance noted by **(A-B)** are based on testing at *p* < 0.05.

In addition to lipid droplet formation in the quail myoblast cell lines by atRA, it was necessary to verify molecular evidence for transdifferentiation of the QM7 cells into adipogenic cells by measuring the expression of critical genes involved in adipogenesis and myogenesis. Expression levels of the zinc finger protein 423 (Znf423) gene, as a marker of an early stage of adipogenic differentiation (Gupta et al., 2010), were significantly increased by the supplementation of 100 μM atRA in 10% CS (Figure 2A). As major adipogenic markers, the expression of Pparγ and fatty acid binding protein 4 (Fabp4) genes were dramatically induced by more than 30-fold with 100 μM atRA compared to other groups (*p* < 0.01) (Figure 2A). In addition, fatty acid transporter 4 (Fatp4), acyl-CoA synthetase long-chain family 1 (Acsl1), and acylglycerolphosphate acyltransferase 1 (Agpat1), which are involved in fatty acid uptake and triacylglycerol (TAG) synthesis, were also significantly up-regulated by the 10% CS with 100 μM atRA compared to the other groups (Figure 2B). On the other hand, the expression levels of myogenic markers, paired box 7 (Pax7), myogenic factor 5 (Myf5), and myogenin (MyoG) were significantly down-regulated by supplementation of 10% CS with 100 μM atRA compared to the other groups (Figure 2C).

### 3.3 Pro-Adipognegic Effect of atRA in Primary Muscle Cells of Quail and Chickens

**FIGURE 3:**
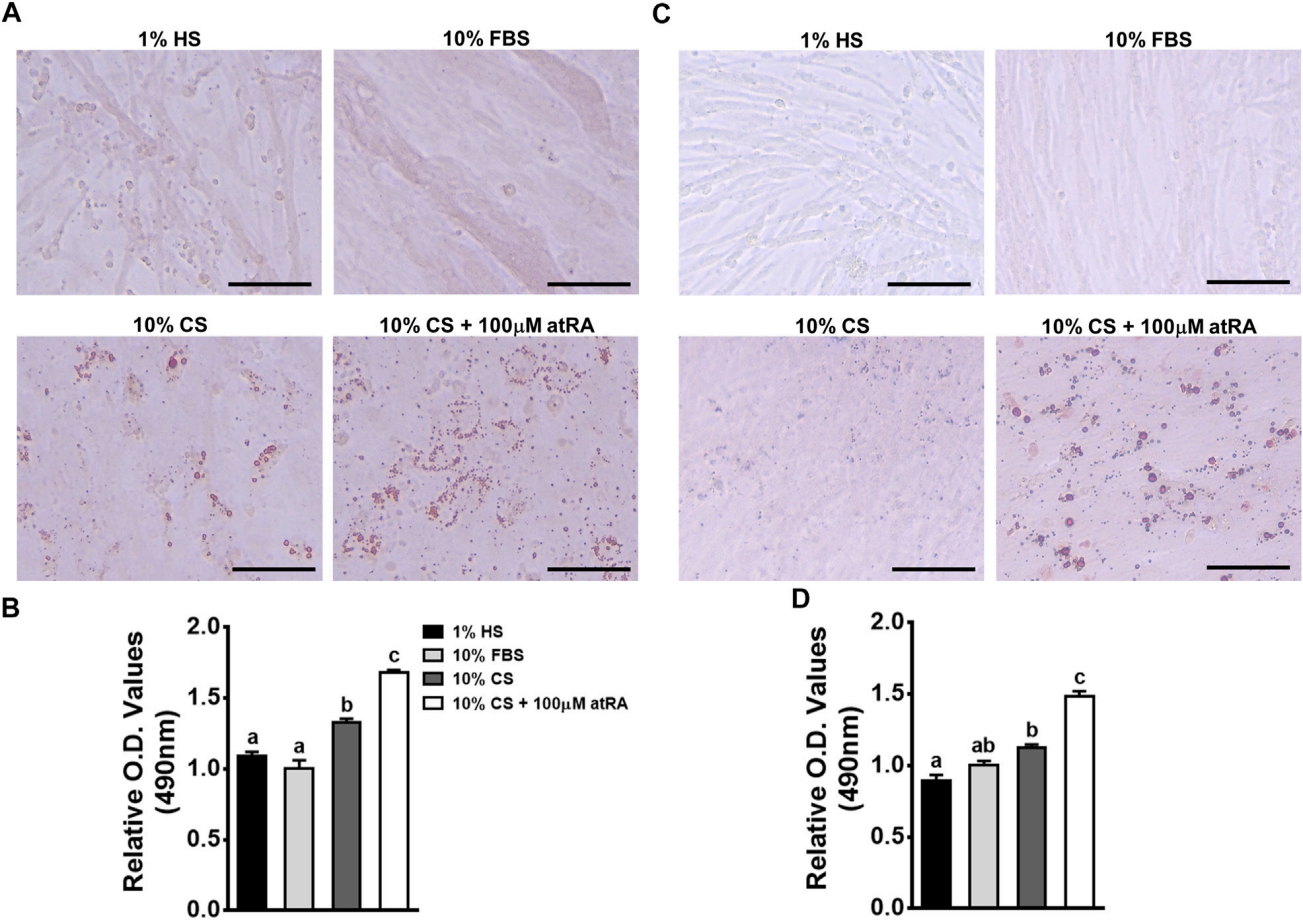
Adipogenic differentiation of avian primary myoblasts by atRA. Primary myoblasts were isolated from breast muscle tissues of quail **(A** and **B)** and chicken **(C** and **D)** embryos at embryonic day 12 and 13, respectively. The cells were grown in four different media for 48 h: 1% HS as a standard myogenic medium, 10% FBS as a standard growth medium, 10% CS as a control medium, and 10% CS with 100 μM of atRA as an adipogenic medium, and then assessed for lipid accumulation. The ORO-stained cells were visualized under a microscope **(A** and **C)** and quantified by a relative amount of ORO per cell which was normalized by a group of 10% FBS **(B** and **D)**. Scale bar: 100 μm. All data were shown as mean ± SEM (*n* = 8 from 4 independent experiments). One-way ANOVA followed by Tukey’s multiple comparison test was used for statistical analysis by the GraphPad PRISM program and statements of significance noted by **(A-C)** are based on testing at *p* < 0.05.

In order to investigate whether the muscle-originated primary cells can be differentiated into adipogenic cells by atRA, isolated primary muscle cells were cultured for 48 h in four different media: 1% HS as a standard myogenic medium, 10% FBS as a standard growth medium, 10% CS as a control medium, and 10% CS with 100 μM of atRA as an adipogneic medium. As expected, primary myocytes isolated from quail and chicken have undergone myogenic differentiation by 1% HS, showing the lining up of myocytes to form nascent myotubes as previously shown in our reports (Shin et al., 2015). Although 10% FBS also resulted in mild degrees of myotube formation, there was no accumulation of lipid droplets in cultures of quail and chicken primary myoblasts (Figures 3A,C). Although fine lipid droplets appeared after 48 h of incubation with 10% CS (Figure 3A), supplementation with 100 μM of atRA seemed to increase sizes and numbers of lipid droplets in cultures of quail and chicken primary myoblasts (Figures 3A,C). These were further confirmed by the finding that amounts of ORO were significantly increased by the supplementation of 100 μM atRA in both quail and chicken primary muscle cells (Figures 3B,D).

### 3.4 Acute Induction of Pparγ Expression in Myogenic Cells by atRA

**FIGURE 4:**
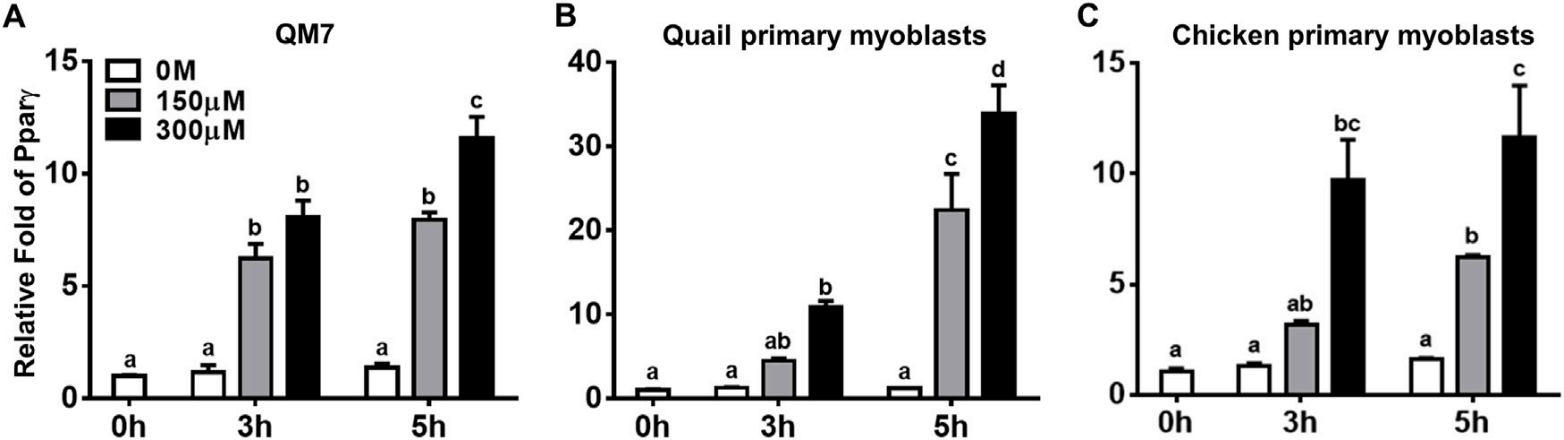
Regulation of Pparγ expression by atRA in myoblasts. Expression levels of Pparγ in QM7 cells **(A)**, and primary myoblasts from quail **(B)** and chickens **(C)** were measured at 0, 3, 5 h after supplementing different concentrations of atRA (0 M, 150 μM, or 300 μM) in 10% CS. Gapdh was used as a housekeeping gene and all expression levels of Pparγ were normalized by the levels of each group at 0 h. All data were shown as mean ± SEM (*n* = 3 from 3 independent experiments). One-way ANOVA followed by Tukey’s multiple comparison test was used for statistical analysis by the GraphPad PRISM program. **(A-D)** indicates statements of significance, *p* < 0.05.

Pparγ, a well-known master regulator for adipogenesis both *in vivo* and *in vitro* (Rosen et al., 1999), is expressed in a few hours after inducing adipogenic differentiation of 3T3-L1 cells (Kim et al., 2014; Ambele et al., 2020). To investigate whether the pro-adipogenic effect of atRA in myogenic cells is accompanied with acute regulation of Pparγ for adipogenic differentiation, expression levels of Pparγ were analyzed at 3 and 5 h after atRA treatment in QM7 cells and primary myoblasts (Figure 4). Pparγ was significantly up-regulated by both 150 and 300 μM of atRA in QM7 cells (5.4-fold and 7-fold, respectively) and by the 300 mM of atRA in quail and chicken myoblasts (8.7-fold and 7.5-fold, respectively) at 3 h compared to the 0 M controls (Figure 4). At 5 h, the expression levels of Pparγ were significantly increased by both 150 and 300 μM of atRA in all three types of cells (5.8-fold and 8.4-fold for the QM7, 18.6-fold and 28.2-fold for the quail myoblasts, and 3.9-fold and 7.3-fold for the chicken myoblasts, respectively, Figure 4). Here, the current study showed acute and dramatic induction of Pparγ mRNA expression in the myoblasts by atRA.

### 3.5 Direct Induction of Pparγ Expression by atRA and Suppression of atRA-Induced Pparγ Expression by an RAR Antagonist in Myoblasts

**FIGURE 5:**
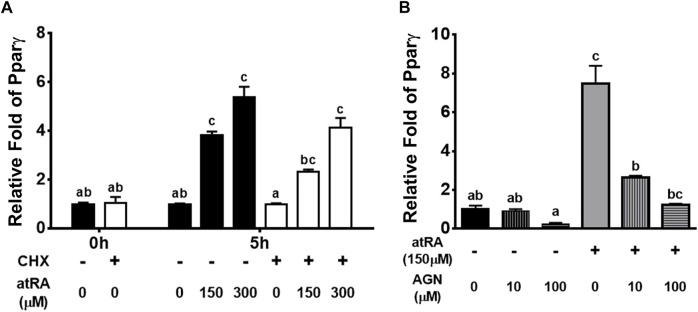
Induction or suppression of Pparγ expression by atRA on the condition of treating cycloheximide or RAR antagonist, respectively, in QM7 cells **(A)** Direct regulation of Pparγ by atRA. Cycloheximide (CHX) was pre-treated for 1 h and then, after washing with PBS, atRA was treated in 10% CS with three different concentrations, 0 M, 150 μM, or 300 μM, for 5 h. The mRNA levels of Pparγ at 1 h after non-treatment of CHX were set as a standard for normalization of relative fold changes **(B)** Suppression of atRA-inducted Pparγ expression by an RAR antagonist. Various concentrations (0 M, 10 μM, and 100 μM) of RAR antagonist (AGN) were treated in 10% CS with 150 μM of atRA. After 5 h, mRNA levels of Pparγ were measured by qPCR, and the group which is 0 M of AGN without 150 μM of atRA was set as a standard for normalization of relative fold changes. All data were shown as mean ± SEM (*n* = 3 from 3 independent experiments). One-way ANOVA followed by Tukey’s multiple comparison test was used for statistical analysis by the GraphPad PRISM program. **(A-C)** indicates statements of significance, *p* < 0.05.

To investigate whether the acute effects of atRA on Pparγ mRNA were due to a direct action on gene transcription or mediated through an increased expression of atRA-dependent auxiliary proteins or transcription factors, we analyzed the effect of atRA in a shorter time, in the presence of the protein synthesis inhibitor CHX (Figure 5A). The expression levels of Pparγ were expectedly increased by atRA at 5 h and those levels were not significantly affected by CHX treatment, showing similar expression patterns at different concentrations of atRA regardless of treatment of CHX (Figure 5A). These results support that atRA-induced Pparγ expression was independent of protein synthesis and thus, a direct downstream event of atRA.

To determine if atRA affects the up-regulation of Pparγ through RAR, QM7 cells were treated with 150 μM of atRA in various concentrations (0, 10 or 100 μM) of AGN, an RAR antagonist, for 5 h. The data showed that AGN dose-dependently decreased atRA-mediated induction of Pparγ expression (Figure 5B), suggesting that RAR is a mediator of atRA-induced Pparγ expression.

### 3.6 Effect of Pparγ Antagonist and Agonist on atRA-Induced Transdifferentiation

**FIGURE 6:**
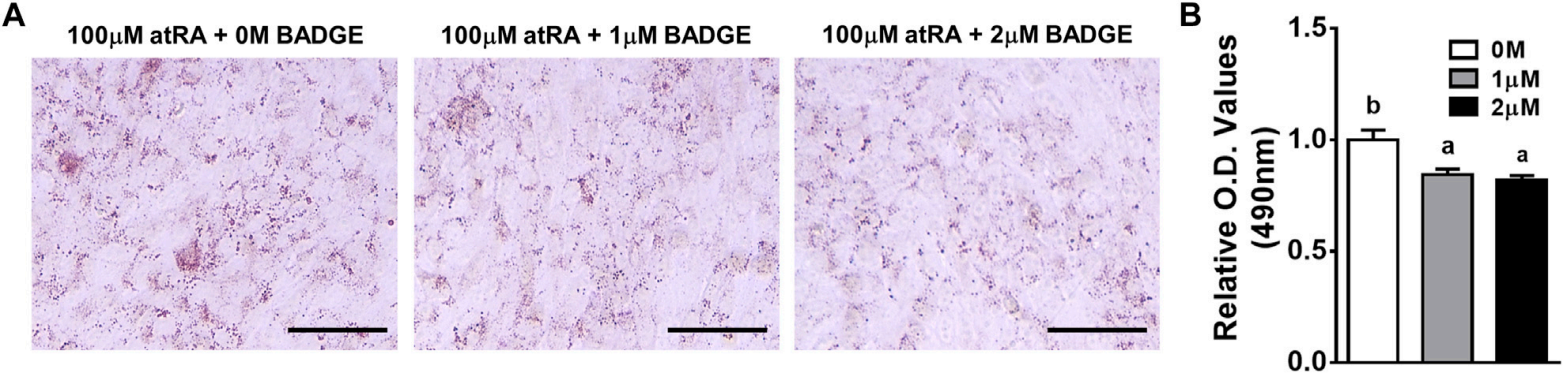
Effects of BADGE as an inhibitor of atRA-induced lipid accumulation in QM7 cells. Oil-Red-O (ORO) staining **(A)** and O.D. values **(B)**. QM7 cells were grown in different concentrations of BADGE (0 M, 1 μM, or 2 μM) in 10% CS with 100 μM of atRA for 2 days, and then assessed for lipid accumulation. Lipid droplets in cells were visualized under a microscope after ORO staining and quantified by a relative amount of ORO per cell which is normalized by a group of the 0 M BADGE. Scale bar: 100 μm. All data were shown as mean ± SEM (*n* = 3 from 3 independent experiments). One-way ANOVA followed by Tukey’s multiple comparison test was used for statistical analysis by the GraphPad PRISM program and statements of significance noted by a-b are based on testing at *p* < 0.05.

Due to the acute expression of Pparγ under the direct effect of atRA, it was investigated whether Pparγ is mediated for atRA-induced transdifferentiation. Utilizing an antagonist (BADGE) of Pparγ, we first examined the effect of BADGE on atRA-induced transdifferentiation. QM7 cells were induced to transdifferentiate for 2 days with 100 μM of atRA in the absence or presence of BADGE (0, 1, or 2 mM). As shown in Figure 6, treatment with BADGE resulted in a significantly reduced lipid accumulation in cells treated with atRA as observed by ORO measurement.

**FIGURE 7:**
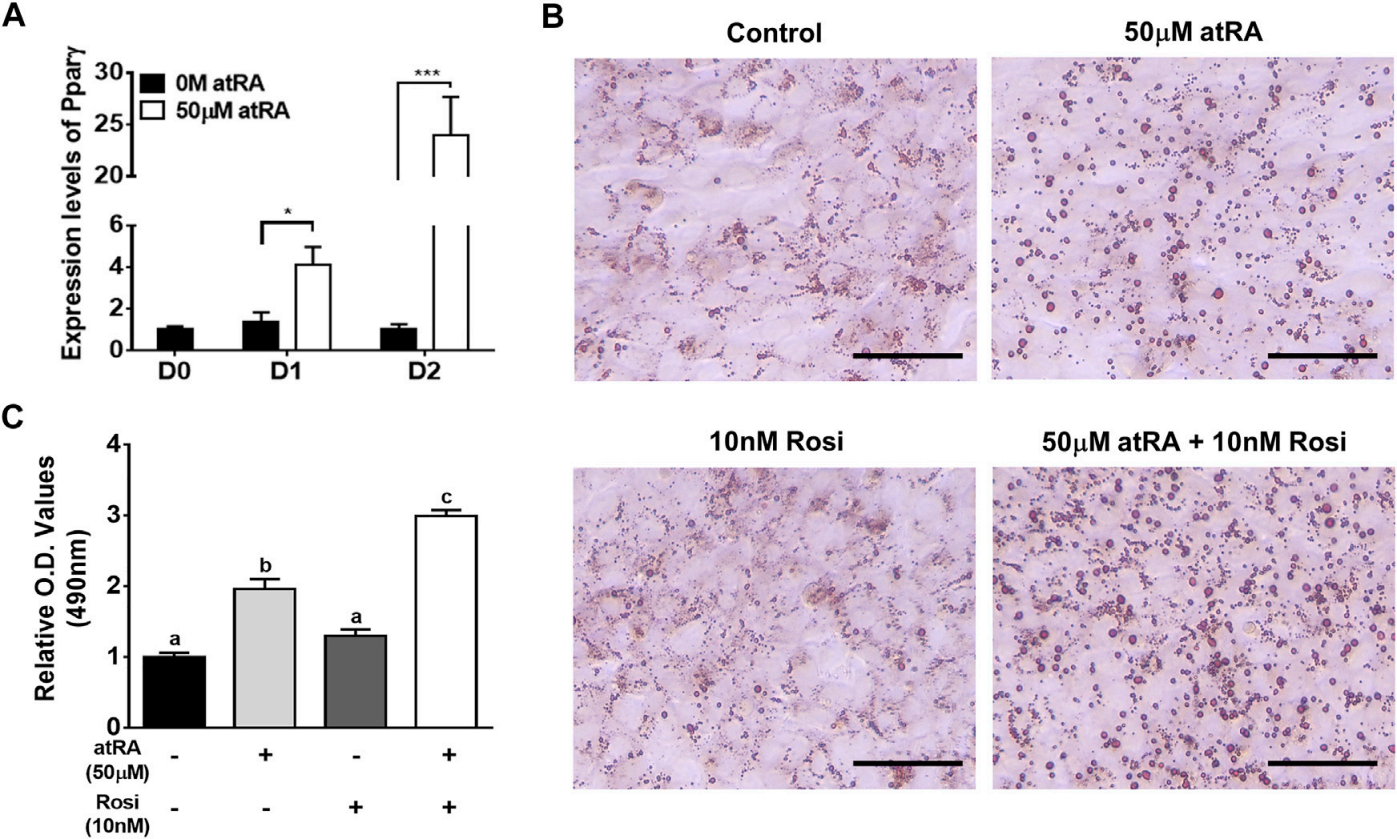
Effect of Rosi as a co-activator on lipid accumulation. Expression levels of Pparγ in QM7 cells **(A)**. Expression levels of Pparγ were measured for 2 days after supplementation of 50 μM of atRA in 10% CS. Gapdh was used as a housekeeping gene and the expression levels are normalized by the levels at day **(D)** 0. *t*-tests were used for statistical analysis between the two groups, the 0 M and 50 μM of atRA, at D1 or D2, respectively, by the GraphPad PRISM 6.02 program. *: *p* < 0.05; ***: *p* < 0.001. Visualization **(B)** and quantification **(C)** of lipid accumulation. QM7 cells were grown in four different media for 2 days: 10% CS as a control media, 10% CS with 50 μM of atRA (50 μM), 10% CS with 10 nM of Rosi, and atRA 10% CS with both 50 μM of atRA and 10 nM of Rosi, both, and then assessed for lipid accumulation. The cells were stained by ORO to visualize lipid droplet accumulation and quantify relative amounts of ORO which is normalized by a group of the 10% CS. Scale bar: 100 μm. One-way ANOVA followed by Tukey’s multiple comparison test was used for statistical analysis by the GraphPad PRISM program. **(A-C)** indicates statements of significance, *p* < 0.05. All data in **(A)** and **(C)** were shown as mean ± SEM (*n* = 4 from 4 independent experiments).

As QM7 cells are a myogenic cell line, Pparγ mRNA expression remained low at all time points of cell culture in the absense of atRA (black bar in Figure 7A). At the low levels of Pparγ expression in the absense of atRA, Rosi, as an agonist of Pparγ, did not significantly affect lipid droplet formation and lipid accumulation compared to a control without Rosi as assessed by ORO staining (Figures 7B,C). Treatment of atRA significantly increased the expression levels of Pparγ by 4-fold or 24-fold at D1 or D2, respectively, compared to the absence of atRA (Figure 7A). As expected, atRA alone induced lipid accumulation by 2-fold in QM7 cells compared to the control cells (Figures 7B,C). Rosi further increased atRA-induced lipid accumulation up to 1.5-fold compared to the cells treated with atRA alone (Figures 7B,C).

## 4 Discussion

Several genetic and nutritional factors have been known to be involved in transdifferentiation of myogenic cells to adipogenic cells *in vitro*. Pparγ and/or C/EBPα, as master regulators of adipogenesis, can induce transdifferentiation of G8 or C2C12 myoblasts onto adipocytes (Hu et al., 1995; Yu et al., 2006). Treatment of 1,25(OH)_2_D_3_, an active form of vitamin D, induced lipid droplet formation on C2C12 myoblasts (Ryan et al., 2013). The current study demonstrated for the first time that a derivative of lipid soluble vitamin A (atRA) can convert both primary myogenic cells and the myoblast cell line to adipogenic cells in avian species.

Although high concentrations (over 1 μM) of atRA inhibit lipid accumulation in adipocytes, adipogenic differentiation of murine preadipocyte cell lines including Ob1771 and 3T3-L1 cells is promoted by atRA (Safonova et al., 1994). In chickens, our recent studies showed that atRA promoted lipid accumulation in primary preadipocytes (Kim D.-H. et al., 2020). In addition, *in ovo* injection of atRA in quails resulted in increased fat pad weight with adipocyte hypotrophy (Kim et al., 2021a). These data suggest pro-adipogenic activity of atRA in preadipocyte and adipose tissues *in vitro* and *in vivo*. Interestingly, it has been recently demonstrated that atRA could induce adipogenesis of non-preadipocytes such as chicken embryonic fibroblasts and fibroblast cell line (DF-1) (Lee et al., 2021). Furthermore, the current study revealed that atRA increased lipid accumulation in cultured avian myoblasts, providing morphological evidence of potential adipogenic transdifferentiation (Figures 1, 3).

In diabetic and/or obese conditions, fine lipid droplets can be formed in muscle fibers, called intramyocellular lipid (IMCL) droplets. One might doubt whether lipid droplets formed by atRA are IMCL droplets found in avian myoblasts rather than transdifferentiated adipocytes. Therefore, it was necessary to investigate whether myoblasts treated with atRA are converted into adipocytes by measuring adipogenic and myogenic marker genes. The current study proved that the expression levels of all selected genes involved in adipocyte determination and differentiation (Znf423, Pparγ, and Fabp4), and fatty acid uptake (Fatp4, Acsl1, and Agpat1) were significantly increased, however, the genes in myogenesis (Pax7, Myf5, and MyoG) were significantly decreased by the supplementation of atRA on QM7 myoblasts. Similar with our study, overexpression of adipogenic factors could convert myogenic cells to adipogenic cells (Hu et al., 1995; Yu et al., 2006), and myogenic factors were suppressed during transdifferentiation of C2C12 cells into mature adipocytes (Teboul et al., 1995; Ryan et al., 2013). Taken together, the morphological and gene expression data, showing inhibition of myofiber formation and promotion of lipid accumulation which are accompanied by the reducing expression of myogenic markers and inducing adipogenic markers, respectively, suggest that atRA could be an inducer of transdifferentiation of avian myoblasts into adipocytes.

As a master regulator of adipogenesis, Pparγ regulates many adipocyte genes involved in lipid metabolism and energy balance (Desvergne and Wahli 1999). Severe lipoatrophy and metabolic disturbance resulted from Pparγ knockout in mice (Jones et al., 2005; Wang et al., 2013) and overexpression of Pparγ in the mouse and pig resulted in increased intramuscular fat with an increased expression of adipogenic markers (Ma et al., 2015; Gu et al., 2021). These genetic models suggest that the ectopic expression of Pparγ in skeletal muscles enhanced lipid accumulation and adipogenic potential. In this study, the expression levels of Pparγ were acutely increased by the supplementation of atRA in avian myogenic cells, suggesting that the initiation of adipogenesis might be achieved by atRA (Figure 4). The acute induction of Pparγ expression by atRA leads to a possibility for direct regulation of Pparγ mRNA expression by atRA. Up-regulation of Pparγ by atRA, regardless of protein synthesis inhibitor (Figure 5A) indicates that atRA can directly increase Pparγ expression. Moreover, it is known that retinol metabolites and analogs function as ligands for RARs (RARα, β, and γ), and/or retinoid X receptors (RXRs α, β, and γ), and heterodimerized RARs/RXRs act as gene transcriptional factors (Gudas and Wagner 2011). Among retinoid metabolites, atRA are mediated by RARs, but not by RXRs (Lehmann et al., 1992). In the current study, up-regulation of Pparγ expression by atRA was significantly and dose-dependently reduced by the treatment of an RAR antagonist, AGN. Taken together, atRA could directly regulate Pparγ expression by binding to RAR to induce lipid accumulation in myoblasts.

The aforementioned findings favor the hypothesis that induced Pparγ expression in myoblasts by atRA can serve as an intermediate regulator of atRA in inducing transdifferentiation of myoblasts. To test the hypothesis, BADGE or Rosi, as antagonist and agonist of Pparγ, respectively, were treated in the absence or presence of atRA. Supplementation of BADGE in the presence of atRA significantly inhibited lipid droplet formations (Figure 6). On the other hand, supplementation of Rosi in the presence of atRA dramatically increased lipid accumulation in QM7 myoblasts compared to the supplementation of atRA alone (Figure 7). However, supplementation of Rosi, when the Pparγ expression was not induced in the absence of atRA treatment, could not increase lipid accumulation in the myoblasts. These data support that the induction of Pparγ expression by atRA is an essential molecular event in myoblasts for atRA-induced transdifferentiation into adipocytes.

So far, it was reported that transdifferentiation of myoblasts to adipocytes could be induced by ectopic expressions of adipogenic transcription factors and treatment with nutritional factors. However, the current study, in avian species, demonstrated first that atRA has effects on inducing transdifferentiation of myoblasts into adipocytes with a direct regulation of Pparγ. Based on the current finding *in vitro*, the challenges for the future are to consolidate the atRA-RAR-Pparγ axis in inducing transdifferentiation of myoblasts into adipocytes and to confirm enhanced marbling fat by atRA *in vivo*.

## Data Availability

The raw data supporting the conclusions of this article will be made available by the authors, without undue reservation.
