# Supplementary material for: Transdifferentiation of Myoblasts Into Adipocytes by All-Trans-Retinoic Acid in Avian
Source: Front Cell Dev Biol. 2022 Apr 6;10:856881. doi: 10.3389/fcell.2022.856881 (PMC9019681; doi:10.3389/fcell.2022.856881)
Supplement: Supplementary file 1 [file Table1.DOCX]

Supplementary Material

**Table S1.** List of primer sequences used in this study.

| Gene | Primer Sequence (5’ to 3’) | | Size (bp) | Accession number |
| --- | --- | --- | --- | --- |
| Znf423 | F: CCAGTGCCCACAGAAGTTCT | R: CCACTGTGCCACCATCAAGT | 124 | XM_025154318.1 |
| Pparγ | F: TGCCAAGCATTTGTATGACTC | R: TGCGAATTGCTACTTCTTTGTT | 200 | NM_001001460 |
| Fabp4 | F: CAAGCTGGGTGAAGAGTTTGATG | R: TCGTAAACTCTTTTGCTGGTAAC | 195 | NM_204290 |
| Fatp4 | F: AAAAGGGGATGCTGCCTATCT | R: GCTTACGCAGCTCCATCTTCT | 374 | FJ868804 |
| Acsl1 | F: GAAGTGAAGCACTTGCTCAAGTG | R: GTTGGTGTCAGCAGGCCAT | 283 | NM_001012578.1 |
| Agpat1 | F: TGTTGCTGTGTATGCTGAGAGTGC | R: TGCCACTAAACTCAAGATCTCAGGGT | 580 | XM_421757.2 |
| Pax7 | F: AGGCCTTTGAGAGGACCCACTA | R: CAGATGGTTGAATGCTGCGAG | 209 | NM_204184.1 |
| MyoG | F: CTGCCCAAGGTGGAGATCCT | R: GGGTTGGTGCCAAACTCCAG | 209 | NM_204184.1 |
| Myf5 | F: TATCACCTGCCGGGACAG | R: AGGCGGTCCACGATGCT | 209 | XM_015857363.2 |
| Gapdh | F: CTCTGTTGTTGACCTGACCTG | R: CAAGTCCACAACACGGTTGCT | 262 | XM_015873412.2 |
