# Supplementary material for: Transdifferentiation of Myoblasts Into Adipocytes by All-Trans-Retinoic Acid in Avian
Source: Front Cell Dev Biol. 2022 Apr 6;10:856881. doi: 10.3389/fcell.2022.856881 (PMC9019681; doi:10.3389/fcell.2022.856881)
Supplement: Supplementary file 2 [file Presentation1.PPTX]

## Slide 1
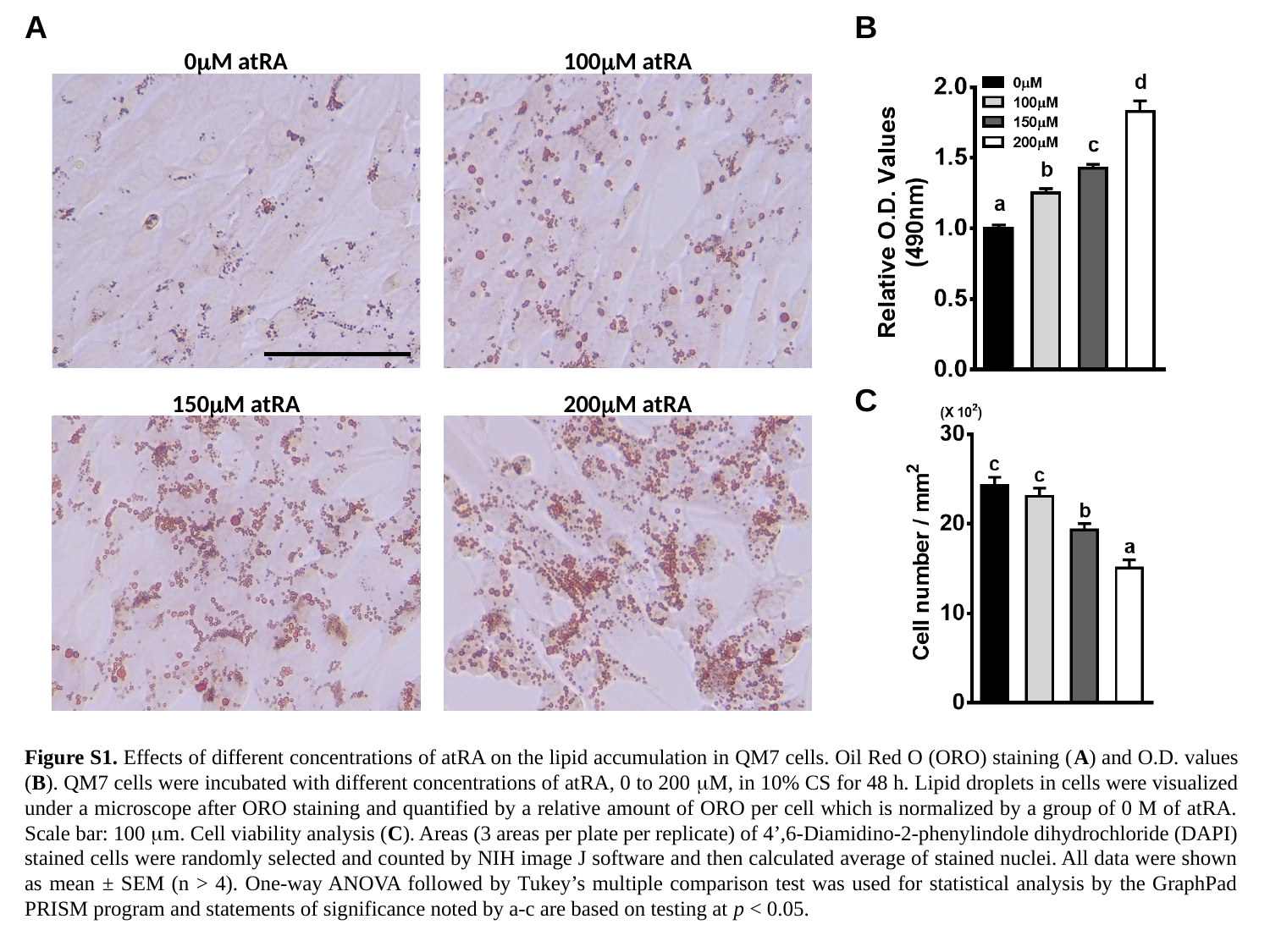

A
B
0mM atRA
100mM atRA
200mM atRA
150mM atRA
C
Figure S1. Effects of different concentrations of atRA on the lipid accumulation in QM7 cells. Oil Red O (ORO) staining (A) and O.D. values (B). QM7 cells were incubated with different concentrations of atRA, 0 to 200 mM, in 10% CS for 48 h. Lipid droplets in cells were visualized under a microscope after ORO staining and quantified by a relative amount of ORO per cell which is normalized by a group of 0 M of atRA. Scale bar: 100 mm. Cell viability analysis (C). Areas (3 areas per plate per replicate) of 4’,6-Diamidino-2-phenylindole dihydrochloride (DAPI) stained cells were randomly selected and counted by NIH image J software and then calculated average of stained nuclei. All data were shown as mean ± SEM (n > 4). One-way ANOVA followed by Tukey’s multiple comparison test was used for statistical analysis by the GraphPad PRISM program and statements of significance noted by a-c are based on testing at p < 0.05.
